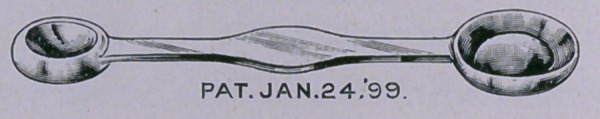# Notes and Notices

**Published:** 1899-04

**Authors:** 


### NOTES AND NOTICES.

Superior to Imported Wines.—Dr. S. F. Howland, of 29 West 42d Street, New York, says: I can say emphatically that I like Speer's Wines far better than any of the imported wines. His Claret and Sauterne are superior dinner wines. His Port beats the world for invalids and weakly persons.

Dr. J. A. Whitman has removed from Beaufort, S. C., to No. 86 Wentworth Street, Charleston, S. C. He will be glad to receive calls from all homœopathists who may be passing through Charleston.

All Physicians Here and in Europe who have used Speer's Port Grape Wine recommend it in preference to any other for its medicinal properties, especially for females, debilitated persons, and the aged.

Examinations of applicants to fill vacancies on the House Staff of the Brooklyn Homœopathic Hospital will be held on April 19th and again on May 8th, at noon, at the Hospital, 109 Cumberland Street.

Henry B. Minton, *Secretary of Committee,*
165 Joralemon Street, Brooklyn, N. Y.

See a lot of men and women in another column carrying on their heads great loads of grapes at Boa Vista vineyards, Portugal, for making into wine. It is interesting. Read all about it. Speer, N. J., wines are made from the same grape, the oldest and finest produced in the world.

The Acme Powder Measure.—Most of the powders used in medicines are, within moderate limits, of practically the same specific gravity, so that weights can be determined by measurement as well as by the troublesome and time-consuming process of weighing out individual powders by the druggist's scales. It is to meet the want of a quick and easy, yet accurate method of determining the bulk requisite for 1, 2, 3, and 5-grain powders, or any combination of these quantities, that this little device was invented. It consists of a centre rod about six inches long, to the opposite ends of which are attached four different sized measures, of the capacity of 1, 2, 3, and 5 grains respectively. It is made of German silver, and heavily silver plated. In using the measure it may be inserted into a bottle or other suitable receptacle containing the powdered substance, and in withdrawing it it is to be pressed or scraped against the side of the receptacle (thus filling the measure level full). It will be found to give surprisingly accurate results as to weights of individual powders, and may be very rapidly performed. The standard taken is sugar of milk, which is a fair average of weight for nearly all medicinal powders, as distilled water is the recognized standard of weight of liquid measure. It occupies no more space than a probe, and can be carried in the pocket case with perfect facility, and will be found extremely practical. Sample sent by mail, including weight table of exceptional substances, on receipt of price, 25 cents.

Sharon Manufacturing Co.,
17 N. Juniper Street, Philadelphia, Pa.

Corsets in Russia.—Hereafter the women of Russia must do without corsets. The Russian Minister of Public Instruction has issued a decree absolutely prohibiting the use of the corset, on the grounds of public health.—*Medical Times*, November, 1898.

## Figures and Tables

**Figure f1:**